# All Signs Point to Cancer: A Case of Cryptic Miliary Tuberculosis in the Setting of Concomitant Cancer

**DOI:** 10.1155/2021/4623086

**Published:** 2021-06-04

**Authors:** Victoria Cress, Sue Min Sophia Kwon, Michael T. Ulrich

**Affiliations:** ^1^Loma Linda University School of Medicine, 11175 Campus Street Loma Linda, Loma Linda 92350, CA, USA; ^2^Loma Linda University Medical Center, Department of Medicine, 1234 Anderson Street Rm. 1503 Loma Linda, Loma Linda 92354, CA, USA; ^3^Riverside University Health System, Department of Medicine, 26520 Cactus Avenue Moreno Valley, Moreno Valley 92555, CA, USA

## Abstract

Dissemination of tuberculosis (TB) is known as miliary tuberculosis. When miliary tuberculosis lacks classic radiographic and clinical features, it can be labeled cryptic miliary tuberculosis and may mimic metastatic cancer. This unusual presentation of an already atypical form of TB often delays diagnosis. We present a case of a 58-year-old female who presented with presumed metastatic carcinoma, who was ultimately diagnosed with both primary breast cancer and disseminated TB. This case emphasizes the need for a high index of suspicion for miliary tuberculosis in patients with presumed or proven malignancy.

## 1. Introduction

Cryptic miliary tuberculosis is a rare presentation of tuberculosis (TB) known as disseminated or miliary TB. Miliary TB accounts for just 2–5% of all TB cases and 8–20% of extrapulmonary TB cases in immunocompetent individuals [1–3]. Multiple organ involvement in disseminated TB can mask classic signs and symptoms and even mimic metastatic cancer; this is known as cryptic miliary TB. In these cases, patients (commonly older) may present with absent or mild fever, progressive wasting, and diffuse lymphadenopathy. This unusual presentation of an already atypical form of TB often delays diagnosis. We present a case of a 58-year-old female who presented with presumed metastatic carcinoma, who was ultimately diagnosed with both primary breast cancer and disseminated TB.

## 2. Case Presentation

A 58-year-old female patient with no pertinent past medical history presented to the emergency department as a transfer from an outside hospital for computerized tomography (CT) findings of retroperitoneal lymphadenopathy with nodularity and stranding of the omental fat, concerning for malignancy/metastatic disease requiring higher level of care for malignancy workup. The patient stated she experienced increasing fatigue, a decrease in appetite, and an unmeasured amount of weight loss over the past month. The patient stated that over the past two weeks, her symptoms had worsened significantly. She endorsed increased thirst, but denied fevers, chills, headache, cough, dyspnea, night sweats, chest pain, or sick contacts. She denied recent travel, stating she had only travelled to Thailand greater than 10 years previously.

On physical exam, the patient was afebrile with stable vital signs. She had clear lung sounds bilaterally, was nontender to abdominal palpation with a negative Murphy's sign, and had an unremarkable cardiac exam. A 2 cm mass in the 4 o'clock position of the left breast was palpated on clinical breast exam.

Laboratory analysis was remarkable for leukocytosis of 13.9x10e9/L, alkaline phosphatase of 310 U/L, and aspartate aminotransferase of 78 U/L. Malignancy workup was obtained: cancer antigen 19–9 and carcinoembryonic antigen were elevated to 301.4 U/mL and 9.0 ng/mL, respectively, with alpha-fetoprotein tumor marker within normal limits. Evaluation for acute and chronic liver pathology was positive solely for hepatitis A, with kidney microsomal antibodies, mitochondrial antibodies, antinuclear antibodies, alpha-1-antitrypsin, and ceruloplasmin all being negative. An iron panel demonstrated findings consistent with anemia of chronic disease (ferritin 2410.3 ng/mL, total iron 47 mcg/dL, and total iron binding capacity 80 mcg/dL). Blood cultures and urine cultures demonstrated no growth. Interferon-gamma release assay (IGRA) for tuberculosis was indeterminate.

The patient received extensive imaging. A CT abdomen/pelvis demonstrated retroperitoneal lymphadenopathy, nodularity and stranding in the omental fat, thickened ring along the periphery of the liver, and cholelithiasis. A CT chest demonstrated mediastinal lymphadenopathy, multiple small nodules scattered in the lungs bilaterally, and a small left pleural effusion. Cardiac bedside ultrasound showed no apparent focal wall motion abnormality and demonstrated adequate systolic left ventricular systolic function with an ejection fraction greater than 60%. Abdominal bedside ultrasound demonstrated multiple gallstones within an enlarged gallbladder measuring approximately 10 cm. Formal right upper quadrant ultrasound demonstrated cholelithiasis with sludge and gallbladder wall thickening as well as a nodular hepatic contour. Magnetic resonance cholangiopancreatography (MRCP) was unable to be obtained due to a history of a bullet wound to the left breast with retained metal fragments. The patient's hepatobiliary iminodiacetic acid (HIDA) scan demonstrated nonvisualization of the bladder supporting cystic duct obstruction and acute cholecystitis. Mammogram showed a 2.5 × 2.7 cm breast mass of the left breast.

The patient was started on Zosyn for cholecystitis and underwent an esophagogastroduodenoscopy (EGD) and colonoscopy which demonstrated nodular gastric mucosa and duodenal erosions. During her hospital course, she underwent multiple biopsies, including an ultrasound-guided core biopsy of her left breast mass, a colon biopsy, a retroperitoneal node biopsy, and a gastric mucosa biopsy. Throughout her hospital course, consultants from hematology/oncology, general surgery, and surgical oncology services informed the patient of the high likelihood of a cancer diagnosis upon pathologic examination of biopsied tissue. However, the patient left the hospital against medical advice before the biopsy results were available.

Upon outpatient hematology/oncology follow-up, the patient received her biopsy results. Her colon biopsy and gastric mucosa biopsy demonstrated no abnormalities. However, her left breast biopsy demonstrated a new diagnosis of cT2N0 grade-2 estrogen receptor-positive, progesterone receptor-positive, and her2-negative invasive carcinoma of no special time with lobular features with a minor component of ductal carcinoma in situ. Her retroperitoneal node biopsy demonstrated lymphoid tissue with necrotizing giant-cell granulomas, positive for mycobacteria on acid-fast bacilli staining. Treatment of the abdominal tuberculosis was to be coordinated by infectious disease and hematology/oncology before undergoing surgical treatment for the breast mass. Upon phone call to confirm an appointment, the patient's husband informed medical staff that the patient had passed away, less than two weeks after her discharge.

## 3. Discussion

Tuberculosis (TB) is a disease that affects people of all age groups in all countries. Due to advances in medical diagnosis and treatment, the worldwide TB incidence in new cases per 100,000 is falling at the rate of about 2% per year [4]. In the setting of impaired cell-mediated immunity, primary TB infection or reactivation of latent TB can result in uncontrolled lymphohematogenous spread of *Mycobacterium tuberculosis*. Immunocompromise such as HIV/AIDS infection, use of immunosuppressive drugs, and organ transplant is a strong risk factor for the development of disseminated TB. Miliary TB accounts for a small percentage of TB infection in otherwise immunocompetent adults. Predisposing factors such as malnutrition, alcohol use, pregnancy, silicosis, and underlying malignancy have been identified [1, 2]. Some studies have revealed a female predominance in clinically unrecognized TB [5, 6].

There is no single pathognomonic sign or symptom for miliary TB; therefore, clinical suspicion must be high to achieve timely diagnosis and treatment. Disseminated *Mycobacterium tuberculosis* spreads to vascular beds of various organs, especially those with relatively high blood flow. Thus, miliary TB's ability to affect any organ system mirrors its variable presentation and elusive diagnosis. Symptoms may be rapidly progressive, episodic, or present with a protracted course. Classic TB symptoms include spiking fevers, chills, night sweats, anorexia, weight loss, and cough. End-organ-specific manifestations of disseminated TB may also offer clues for diagnosis, such as choroidal tubercles in the eye, cutaneous lesions, and TB meningitis. Tuberculous lymphadenitis presents with diffuse, nontender lymphadenopathy [1–3, 7, 8]. These symptoms are also highly suspicious for malignancy which can further obscure the definitive diagnosis. Diagnosis may be masked by more classic pathologies such as chronic obstructive pulmonary disease (COPD) exacerbation and pulmonary thromboembolism [5]. Rarely, miliary TB can become fulminant leading to acute respiratory distress syndrome (ARDS), septic shock, multiorgan failure, and death [8, 9].

The term “miliary tuberculosis” is derived from the disease's classic presentation on chest radiograph, resembling millet seeds. However, typical chest radiographic findings may be subtle or absent until late in the disease course. In many cases, miliary TB and especially its cryptic presentation are only diagnosed upon autopsy [5, 6]. Advances in diagnosis tools such as high-resolution computerized tomography (CT) and magnetic resonance imaging (MRI) have allowed for dramatic increases in antemortem diagnosis and are especially useful for identifying extrapulmonary miliary lesions such as in the mesentery, lymph nodes, liver, and spleen [1, 2]. Despite these advances in clinical imaging, difficulty in differentiation between TB lesions and malignant tumors based on clinical and radiological presentation continues [5]. Miliary TB lesions also take up fluorine-18 fluorodeoxyglucose (F-18 FDG) radiotracer on positron emission tomography (PET) CT scan which can either aid in defining extent of known dissemination or can further mimic malignancy and postpone definitive diagnosis [2, 7].

Affected patients may present with nonspecific normochromic anemia, leukopenia or leukocytosis, and elevated erythrocyte sedimentation rate (ESR). When clinical suspicion arises, additional tests may offer further advances toward definitive diagnosis. Testing for TB with skin testing and interferon-gamma release assay (IGRA) is the first of these. IGRA is preferred in those likely infected due to ease of sample acquisition and since a negative TB skin test is common in disseminated disease [1, 8, 10]. A positive IGRA cannot distinguish between active and latent TB, but a negative result may be helpful in ruling out miliary TB. Definitive diagnosis comes from tissue and fluid sampling. For adults with suspected miliary or extrapulmonary TB, sputum samples, fine needle aspirates, and other collected body fluids should be sent for acid-fast bacillus smear microscopy, mycobacterial culture, nucleic acid amplification testing, and histological examination [2, 10]. Culture isolates from mycobacterial culture-positive samples may also be submitted for genotyping and drug susceptibility on an as-indicated basis.

Active TB infection is typically treated with rifamycin, isoniazid, pyrazinamide, and ethambutol for 2 months followed by rifamycin and isoniazid for 4 months, for a total of 6 months of treatment. Most patients with miliary TB receive this empiric treatment and some have shown further improvement with the use of steroids [3, 9, 11]. However, there is no current consensus regarding the optimum duration of treatment for disseminated TB or the use of steroids in treatment [2].

Often in cases of cryptic miliary TB, the suspicion of TB arises too late or not at all. This may be because miliary TB diagnosis is often being overlooked in countries with low incidence of TB [5, 6]. Despite available diagnostic tools and treatment, mortality of miliary TB is still 25–30% in adults [1, 2]. This is often related to delay in diagnosis and subsequent delays in treatment, further emphasizing the need for high clinical suspicion and clinician education of the presentation and appropriate diagnostic steps. Further studies on definitive early diagnosis of disseminated TB and the most effective treatment regimens are also indicated.

## 4. Conclusions

- Miliary tuberculosis is a dramatically variable disease that requires a high index of suspicion and can present insidiously.
- Routine tuberculosis testing in patients presenting with presumed metastatic malignancy may prove beneficial.
- Further studies are needed to determine the most effective treatment regimen and duration for disseminated tuberculosis.

## References

[1] Sharma S. K., Mohan A., Sharma A., Mitra D. K. (2005). Miliary tuberculosis: new insights into an old disease. *The Lancet Infectious Diseases*.

[2] Sharma S. K., Mohan A., Sharma A., Manget J. J. (2012). Challenges in the diagnosis & treatment of miliary tuberculosis. *Indian Journal of Medical Research*.

[3] Chamberlin K., Orfanos S., Mukherjee A., Moy E., Koganti M., Khan W. (2018). A case of disseminated tuberculosis mimicking metastatic cancer. *Respiratory Medicine Case Reports*.

[4] Organisation mondiale de la santé (2018). *Global Tuberculosis Report 2018*.

[5] Savic I., Trifunovic-Skodric V., Mitrovic D. (2016). Clinically unrecognized miliary tuberculosis: an autopsy study. *Annals of Saudi Medicine*.

[6] Vasankari T., Liippo K., Tala E. (2003). Overt and cryptic miliary tuberculosis misdiagnosed until autopsy. *Scandinavian Journal of Infectious Diseases*.

[7] Das C. J., Kumar R., Balakrishnan V. B., Chawla M., Malhotra A. (2008). Disseminated tuberculosis masquerading as metastatic breast carcinoma on PET-CT. *Clinical Nuclear Medicine*.

[8] Golden M. P., Vikram H. R. (2005). Extrapulmonary tuberculosis: an overview. *American Family Physician*.

[9] Barman B., Tiewsoh I., Lynrah K., Wankhar B., Beyong T., Issar N. (2017). Miliary tuberculosis with pulmonary and extrapulmonary component complicated with acute respiratory distress syndrome. *Journal of Family Medicine and Primary Care*.

[10] Lewinsohn D. M., Leonard M. K., LoBue P. A. (2017). Official American thoracic society/infectious diseases society of America/centers for disease control and prevention clinical practice guidelines: diagnosis of tuberculosis in adults and children. *Clinical Infectious Diseases*.

[11] Ali Chaudhry L., Al-Solaiman S. (2013). Multifocal tuberculosis: many faces of an old menace. *International Journal of Mycobacteriology*.

